# The Medical Law of Maryland

**Published:** 1894-05

**Authors:** 


THE MEDICAL LAW OF MARYLAND has been re-enacted with additions and amendments as follows:

That every license to practice medicine and surgery, issued pursuant to the provisions of this Act, shall be subscribed by the president and secretary of the board before whom the applicant has passed. It shall also have affixed to it by the person authorized to affix the same the seal of said medical and chirurgical faculty of Maryland, or of the Maryland State Homœopathic Medical Society, as the license may require, every such license to be in the following form, and to the following effect:

*To All Whom it May Concern, Greeting:*

Be it known, that on the day of , A. D., having offered us satisfactory proof that was more than twenty-one years of age, and had received a proper preliminary education, we therefore give a written order for the examination of said before one of the board of medical examiners of the State of Maryland; that the said was fully examined before our said board and found proficient, and qualified to practice medicine and surgery, we, therefore, have granted to said this our license to practice medicine and surgery in the State of Maryland as a physician and surgeon, and have caused the names president and secretary of our board to be subscribed and the seal of our society to be affixed thereto.

Witness our hands and the seal of our said society, this day of A. D.

President.

Secretary.

[SEAL OF SOCIETY.]

From and after the first day of July, 1894, no person shall practice medicine or surgery in the State of Maryland, unless he or she shall be duly registered as a physician or surgeon, in accordance with the provisions of this Act.

Every person who was practicing medicine in the State of Maryland, on or before the first day of June, 1892, shall be entitled to be registered as physician or surgeon, or both, upon application to the clerk of the

Circuit Court of the county in which he or she may reside or to the clerk of the Circuit Court of Baltimore City, if the applicant shall reside in Baltimore City, and such application shall be in writing, signed by the applicant, who shall state, under oath, that he or she was in fact a practitioner of medicine or surgery, in the State of Maryland, on or before the first day of June, 1892, and thereupon it shall be the duty of said clerk to register such application and the name of such applicant, as physician or surgeon, or both, in a book to be kept for such purpose, and a certified copy of such entry of registration, under the seal of the Court, shall be legal evidence of such registration in all the courts of the State.

All persons who have commenced to practice medicine or surgery, in the State of Maryland, since the first day of June, 1892, or who shall hereafter commence to practice medicine or surgery in this State shall not be entitled to be registered in the Registry of Phvsicians and Surgeons, as required by law, except upon filing with the clerk of the Circuit Court, of the county or city, in which he or she shall reside, a license from one of the duly constituted Boards of Medical Examiners of this State, in accordance with the terms of Sections 47 and 48 of this article, except that physicians and surgeons who have come into this State since said first day of June, 1892, or who shall hereafter come into this State to follow the practice of medicine and surgery, may receive a license, which shall entitle them to be registered as physicians and surgeons, in accordance with law, upon application to one of the duly constituted Boards of Medical Examiners, in accordance with the provisions of Section 56 of this act.

Physicians and surgeons of good moral and professional standing, who have come into this State with intent to follow the practice of medicine and surgery within this state, since the first day of June, 1892, or who shall hereafter come into this State, being graduates of a medical college or university, of good standing, and who have been practitioners of medicine or surgery for more than ten years prior to the date of the application may make application to the president of one of the boards of medical examiners of this State, which application shall be under oath, and shall state where and how long said applicant has been engaged in the practice of medicine and surgery, and from what medical college, university or other institution of learning, he or she has graduated; and thereupon the said board of medical examiners shall have the authority and discretion to require said applicant to undergo an examination, in accordance with the provisions of Sections 41 to 47 inclusive of this article, or may require said applicant to submit to a special examination, the terms and methods of which shall be prescribed by the said board of medical examiners, and upon paying the fee for examination, as set out in Section 45 of this article, after the examination and determination of said board thereupon that said applicant is qualified to practice medicine and surgery, and that he is entitled to a license, a license shall be issued to him to the same effect as the form of license set out in section 47 of this article; which license shall then be filed and recorded as provided by section 47 of this article and it shall then be the duty of the clerk of the court to register the name of the person so licensed as physician or surgeon, or both, in accordance with the provisions of this Act.

All persons whose licenses have been heretofore filed and recorded in accordance with section 48 of this article shall be held to be duly registered physicians and surgeons within the provisions of section 53 of this article, and all persons who shall hereafter receive and file licenses to be recorded in accordance with said section 48, shall be registered as physicians and surgeons under said section, and the fee to be paid for such registration and for the registration of the application to the clerk, or the license therewith as the case may require, shall be one dollar.

If any person shall unlawfully obtain and procure himself to be registered as physician or surgeon, either by false and untrue statement contained in his application to the clerk of this court, as required by this article, or by presenting to said clerk a false or untrue license, or one fraudulently obtained by false and fraudulent statements made to one of said boards of medical examiners, he or she shall be deemed guilty of a misdemeanor and shall be fined not less than fifty dollars or more than five hundred dollars, and shall forfeit all rights and immunities obtained or conferred upon him by virtue of such registration, as physician or surgeon.

Any person who after the 1st day of July, 1894, shall practice or attempt to practice medicine or surgery in this State without being registered in accordance with the provisions of this Act shall be guilty of a misdemeanor, and shall be fined not less than ten dollars nor more than two hundred dollars for each offense.

The provisions of this Act shall not apply to any midwife or person who may render gratuitous services in case of emergency.

